# Is there an epilepsy belt of high prevalence rate in China?

**DOI:** 10.3389/fneur.2022.994305

**Published:** 2022-10-19

**Authors:** Li Wu, Zeming Ye, Youfu Li, Ming Luo, Shaojun Li, Hongyan Mi, Zhihua Qiu, Yongjun Jiang

**Affiliations:** ^1^Department of Neurology, The Second Affiliated Hospital of Guangzhou Medical University, Guangzhou, China; ^2^Guangzhou Panyu District Hexian Memorial Hospital, Guangzhou, China

**Keywords:** China, epilepsy, prevalence, epidemiology, risk factors

## Abstract

**Background:**

Epilepsy is one of the leading neurological diseases. Our study is aimed to determine whether there is a focal region of high epilepsy prevalence in China.

**Methods:**

All studies published between 1981 and 2020 investigating the prevalence of epilepsy in China were systematically reviewed. The geographical location, sample size, number of cases, urbanization rate, gross domestic product (GDP) per capita, percentage of <15 years old, and medical insurance per capita were derived and analyzed. Criteria for a provincial region of high prevalence was defined as with higher epilepsy prevalence than the average prevalence of epilepsy in China.

**Results:**

A total of 60 studies provided data on the prevalence of epilepsy in 29 of 33 provincial regions of China. The average prevalence in China was 1.68 per 1,000, and 12 provincial regions met our criteria for a region of high epilepsy prevalence and constitute an epilepsy belt ranging along the division between the second step and the third step of China. The prevalence in the epilepsy belt was 331.9 per 100,000 population compared with 125.3 per 100,000 in regions outside the belt (*P* < 0.05). Surprisingly, there was no significant difference in sample size, number of cases, urbanization rate, GDP per capita, percentage of <15 years old, or medical insurance per capita between the regions in and outside the epilepsy belt.

**Conclusions:**

An epilepsy belt of high prevalence exists in 12 provincial regions locating along the division between the second step and the third step of China.

## Background

Epilepsy is one of the commonest chronic neurological disorders, affects people of all races, ages and locations in the worldwide and causes severe burden to society. After a long period of repetitive seizures, patients probably would suffer from sense of shame on epilepsy, decline of intelligence and social viability, and increased suicide rate and unintentional mortality. The global death due to epilepsy increases from 130,200 to 177,600 from 1990 to 2010 (1). In addition to the high mortality, epilepsy also induces disability, which is reflected by the remarkable years lived with disability (YLDs) and disability-adjusted life-years (DALYs). The possible reason is that a large portion of patients with epilepsy are young patients. It is estimated that there are 70 million patients with epilepsy in the world while the incidence and prevalence of epilepsy are different among different regions (2). People in poor regions are prone to have epilepsy with the prevalence of 10–40 per 1,000 in developing countries like sub-Saharan Africa and 4.9 per 1,000 in developed countries like Europe.

China has achieved great success in the economy in the last four decades since 1978, the year the policy of Reform and Open up was established. Thanks to the economic miracle, China has made enormous strides in health improvement like the longer life expectancy (60.4 in 1970 to 72.9 in 2010 for male; 63.5 to 79.0 for female). Unlike the increased mortality in the worldwide, the death caused by epilepsy has decreased by 34.8% from 1990 to 2010 (from 187,000 to 122,000) (3). The DALYs has also decreased from 1,723,100 to 1,597,400. A recent systematic review summarized the variable epilepsy prevalence in different regions of China and analyzed the possible issues (4). As the authors pointed out, the geography may contribute to the prevalence of epilepsy since there are tremendous differences of economic development, ethic, and medical policy among different regions in China Mainland. Previous studies have suggested that stroke belt, another common neurological disorder, exists in United States (5) and China Mainland (6). Thus, we hypothesized that there may be an epilepsy belt in China as many individual studies have shown the geographic variation of epilepsy prevalence.

## Methods

### Search strategy

We performed the search on the international databases PubMed, ISI Web of Science, Scopus, and Embase, and the Chinese databases China National Knowledge Infrastructure (CNKI) and Chinese Wanfang Data from the established date to December 2020 using the terms “epilepsy AND (prevalence OR epidemiology) AND (China OR Chinese).” Relevant studies were also checked from the reference lists.

### Selection criteria

Publications met the following criteria were used for further analysis: (1) Complete population-based case ascertainment in China. (2) Publications provided the prevalence of epilepsy. (3) Epilepsy diagnosis was made according to the criteria of the World Health Organization (WHO) or International League against Epilepsy (ILAE) or the local guideline. (4) Studies were published in Chinese or English. The studies were excluded if they were sampled in specific areas like a hospital. Moreover, if two or more publications reported the same study of the same population, only one publication would be included.

### Classification of geographical regions

As the third largest country in the world, China has an area of 9.6 million square kilometers and a coastline of 18,000 km. It spans ~50 degrees of latitude and 62 degrees of longitude, and includes plains, basins, plateaus, foothills, and mountains. In which rugged plateaus, foothills and mountains occupy nearly 70% of the whole land, and form a topographic feature higher in the West and lower in the East like a three-step ladder (7). Base on locations, it has been divided into 34 provincial regions, which contains 23 provinces (Taiwan included), five autonomous regions, four municipalities and two special administrative regions (Macao and Hong Kong) (8). In this study, Chongqing was merged into Sichuan Province, since Chongqing had been a city in Sichuan Province until 1997, and some of the researches used were conducted before 1997. In total, 29 out of 33 provincial regions were included.

### Sources of data for epilepsy prevalence

If only one study was available for the prevalence of epilepsy in a provincial region, the single-source was accepted. If more than one eligible study were available for the prevalence of epilepsy in a provincial region, the prevalence rates from all sources were combined to produce an average rate which was weighted according to the sample size. If no study was available for the prevalence of epilepsy in a provincial region, the prevalence rate of this provincial region was noted as N/A.

### Illustration of the survey methods

In most of the studies we included, the investigators used a random cluster sample survey method to select the cluster where they would go to do the following survey, with a street, village or town in a specific region constituting one cluster. During the following epidemiological survey (mostly door-to-door survey), the residents answered a written questionnaire with the help of well-trained investigators, and then were evaluated by board-certified neurologists at home or in hospitals (9, 10).

Clue investigation was mainly used in vast rural areas where door-to-door survey was impractical. It was a community-based screening strategy among defined population which required employed well-trained local physicians or health workers to collect all clues available to discover patients within a study period (11–13).

One-stage detection method was a community-based screening method which gathered participating residents in the place of screening service activity (a community center or other high-traffic area), where the residents answered a written questionnaire with the help of well-trained social workers, and then were evaluated by board-certified neurologists at the same place (14).

### Geographical classification of epilepsy prevalence and definition of the epilepsy belt

Provincial regions with epilepsy prevalence higher than the average epilepsy prevalence in China were classified as regions with high epilepsy prevalence. The epilepsy belt was defined as a region containing provincial regions with high epilepsy prevalence.

### Exploring possible causes of the epilepsy belt

To explore the possible causes of the epilepsy belt, we reviewed Chinese nationwide epidemiological surveys to obtain the prevalence of risk factors for epilepsy, such as poverty, young age and countryside. Urbanization rate, GDP per capita, percentage of <15 years old and per capita medical insurance in 2018 were retrieved from China Yearbook of Statistics and China Yearbook of Health Statistics. To increase homogeneity, we did not combine the data from multisource, only those from the most representative survey were analyzed. If only 1 source was available for the data of a given factor, the single-source was accepted. If multiple sources were available for the data of a given risk factor, the survey with larger sample size was selected.

### Data extraction

Publications were appraised by two independent investigators (LW and ZY) and any disagreement was settled by the third investigators (YL). The information of the studies, i.e., the first author's name, year of survey, geographic location, age limitation, sample size, gender of population, urban or suburban, and case size, has been extracted.

### Statistical analysis

Epilepsy prevalence in the 33 provincial regions of China including Hong Kong, Macao, and Taiwan was analyzed. Crude epilepsy prevalence rates were reported as the number of cases of epilepsy at the research point per 1,000 individuals. After determining the epilepsy belt, the overall epilepsy prevalence in the epilepsy belt was calculated from the corresponding provincial prevalence rates by weighting the provincial population size. Distribution of risk factors for epilepsy, such as urbanization rate, GDP per capita, percentage of <15 years old and per capita medical insurance in provincial regions, was obtained from nationwide epidemiological surveys. Using these provincial data, distribution of risk factors in epilepsy belt and regions outside was calculated by weighting the provincial population size. Provincial epilepsy prevalence and possible influential factors in population were ranked, with their relationship analyzed by Spearman rank correlation. Differences in risk factor distribution between epilepsy belt and regions outside were compared using 1-way ANOVA, with provincial data treated as unit of analysis. A *P* < 0.05 was defined as statistical significance.

## Results

### Results of literature searching and quality of the included studies

**Figure 1:**
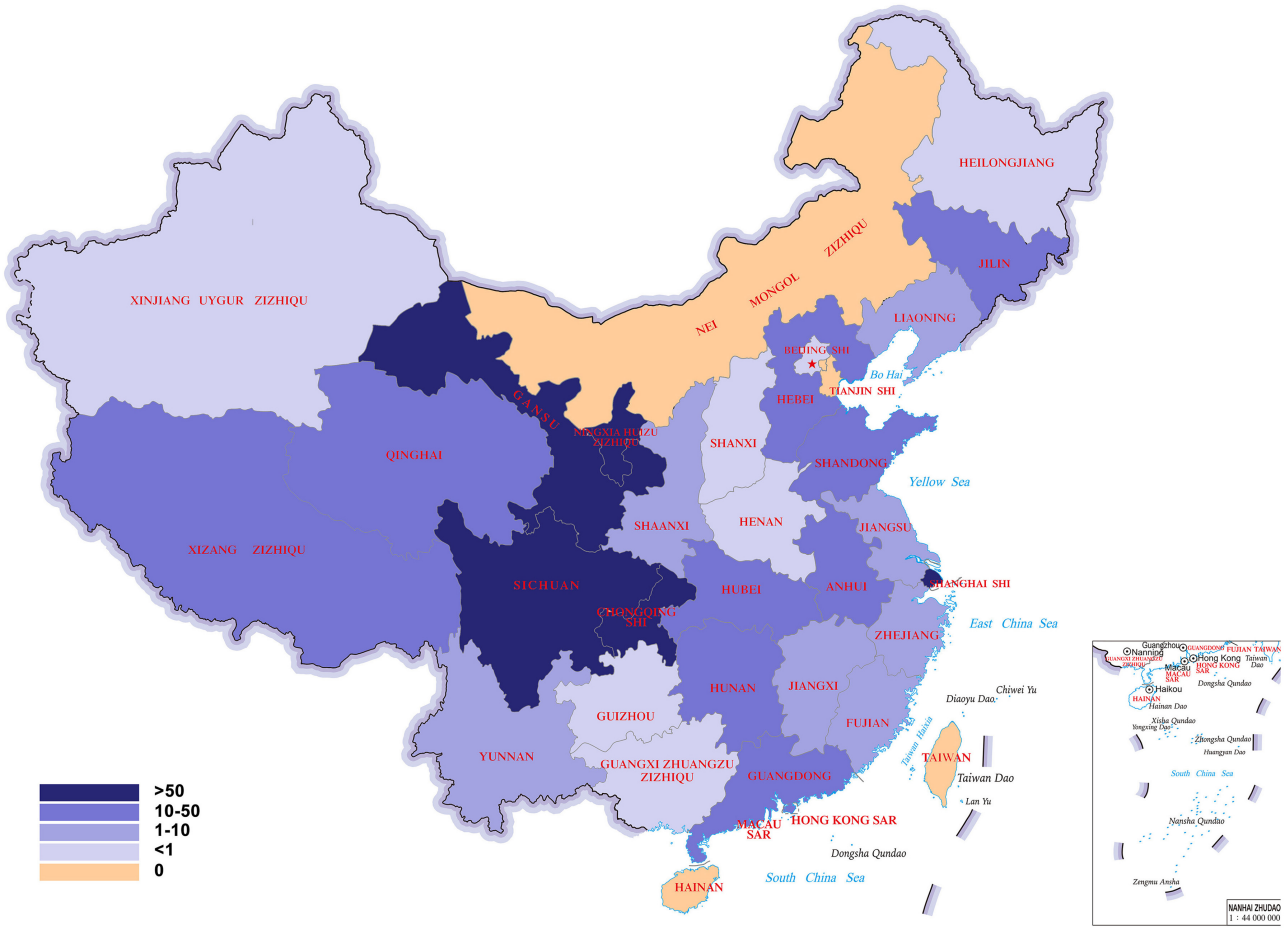
Sampling rates of the 29 provincial regions from the enrolled 60 studies were shown. The sampling rates [(sampling size/numbers of population in 2018) × 1,000‰] were distinct across the country, higher in central area and Southeast regions and lower in West regions.

The database search for studies on epilepsy prevalence in China yielded 456 articles. The abstracts of these articles were screened and in which 395 did not meet the eligibility criteria. The remaining 56 papers have been read thoroughly, and the relevant references have been tracked, an additional four publications have identified. Eventually, 60 studies (60 publications) met the selection criteria for eligibility and had been analyzed. Table 1 showed the characteristics of these included studies. The 60 eligible studies provided data for the prevalence of epilepsy in 29 (87.88%, including Hong Kong) of the 33 provincial regions in total. In the 60 included studies, 39 of them used door-to-door survey method. The diagnosis of epilepsy was confirmed by specialized doctors. For epilepsy diagnostic criteria, 38 of 60 studies used WHO criteria or ILAE criteria, and the other 22 studies used local guideline criteria. The sampling rates of the 29 provincial regions from the enrolled 60 studies were shown in Figure 1, and were distinct across the country, higher in central area and Southeast regions and lower in West regions.

**Table 1:** Characteristics of the included epidemiological surveys on epilepsy prevalence in China.

**First author**	**Publication time**	**Survey time**	**Survey method**	**Diagnostic criteria**	**Provincial regions**	**Urban/ Rural**	**Age range**	**Sample size (*N*)**	**Case (*N*)**	**Male (*N*)**	**Male case (*N*)**	**Prevalence (per 1,000)**
Zhou (15)	1981	1980.03–1980.06	Not mentioned	Local	Shanghai	Urban	All	751,594	531		196	0.71
Gao (16)	1982	1979.04–1979.06	Door-to-door	Local	Shanghai	Both	All	409,876	734		374	1.79
Xu (17)	1982	1978.01–1979.12	Not mentioned	Local	Liaoning	Rural	All	66,595	109	33,497	64	1.64
Wang (18)	1984	1981.05–1981.07	Door-to-door	WHO	Beijing	Urban	All	11,493	44	5,530	23	3.83
Tong (19)	1986	1984.01–1984.12	Not mentioned	WHO	Jiangsu	Rural	All	12,063	30	5,782	11	2.49
Chen (20)	1986	1984.01–1984.12	Door-to-door	Local	Shaanxi	Rural	All	10,038	67		28	6.67
Zhang (21)	1986	1985.02–1985.05	Door-to-door	WHO	Zhejiang	Rural	All	11,894	19	5,983	11	1.60
Zhang (22)	1986	1983.10–1985.02	Not mentioned	Local	Shandong	Rural	All	600,000	2,125		1,163	3.54
Wang (23)	1987	1983.01-	Door-to-door	WHO	Hunan	Urban	All	10,993	46	96,776		4.14
Kong (24)	1987	1983.01-	Door-to-door	WHO	Ningxia	Urban	All	10,641	44			4.13
Huang (25)	1987	1979.05–1979.06	Door-to-door	Local	Guangdong	Both	All	1,212,692	1,490	618,958	798	1.23
Yao (26)	1987	1985.01–1985.03	Door-to-door	Local	Guangxi	Rural	All	11,201	38	5,612	25	3.39
Lu (27)	1987	1985.01–	Door-to-door	WHO	Hunan	Rural	All	15,308	79			5.16
Wu (28)	1989	1987.05–1987.08	Door-to-door	WHO	Hunan	Rural	All	25,085	32	12,903	18	1.28
Liu (29)	1990	1987.05–1987.09	Door-to-door	WHO	Shaanxi	Both	All	160,263	144	80,789	85	0.90
Yang (30)	1990	1984.04–1987.07	Door-to-door	ILAE	Shaanxi, Gansu, Ningxia, Qinghai, Xinjiang	Both	0–14 years	37,003	35			
								36,405	60			
								12,095	12			
								29,421	49			
								20,236	31			
								135,160	187	69,393	118	1.55
He (31)	1991	1987.05–1987.09	Door-to-door	Local	Yunnan	Urban	All	68,551	39		20	0.57
Tang (32)	1991	1987.05–1987.09	Door-to-door	Local	Jiangsu, Anhui, Zhejiang, Jiangxi, Fujian	Both	≥20 years	114,102	63			0.55
								106,335	33			0.31
								102,186	20			0.20
								108,715	22			0.20
								107,154	22			0.21
								538,492	160	275,741	89	0.29
Shan (33)	1992	1988.05–1988.09	Door-to-door	Local	Jiangsu	Both	0–14 years	12,259	58	6,323	33	4.73
Sun (34)	1992	N/A	Door-to-door	WHO	Yunnan	Both	All	11,359	45	5,549		3.96
Li (35)	1993	1986.01.01–1986.12.31	Not mentioned	Local	Shanxi	Both	All	53,720	40	26,338	25	0.74
Liang (36)	1993	1987.04–1987.08	Door-to-door	WHO	Shandong	Both	All	699,268	589		350	0.84
Xiao (37)	1993	1986–1987	Not mentioned	Local	Sichuan	Both	All	126,876	336			2.65
Tao (38)	1994	1987	Door-to-door	WHO	Guizhou	Urban	All	10,173	20	4,963	10	1.97
Wang (39)	1994	1992.03–1992.07	Door-to-door	WHO	Hunan	Rural	All	43,330	52	21,774	30	1.20
Bai (40)	1996	N/A	Door-to-door	Local	Shandong	Both	All	176,530	151		82	0.86
Da (41)	1997	1987.08–1987.11	Door-to-door	WHO	Tibet	Rural	All	31,423	76	15,489	36	2.42
Zhang (42)	1997	1996.05–1996.10	Door-to-door	WHO	Hunan	Rural	All	806,254	1,358			1.68
Li (10)	1997	1987	Door-to-door	WHO	Qinghai	Both	All	101,582	145			1.43
Ren (12)	1997	N/A	Clue investigation	Local	Hebei	Rural	All	46,998	34			0.72
Zhan (43)	1999	1992.12–1993.12	Not mentioned	ILAE	Sichuan	Rural	All	43,992	128	22,902	81	2.91
Hong (44)	2000	1998.01.01–	Door-to-door	WHO	Zhejiang	Rural	All	88,515	172		107	1.94
Du (45)	2002	2000.10–2000.12	Door-to-door	WHO	Ningxia	Rural	All	11,630	99	6,000	45	8.51
Huang (46)	2002	2000	Door-to-door	Local	Shanghai	Rural	All	48,628	151	22,902	81	3.11
Zhang (47)	2002	2000.10–2001.03	Door-to-door	ILAE	Jiangsu	Rural	0–14 years	1,589	6	791	4	3.78
Fong (48)	2003	2002	One-to-one interviews	ILAE	Hongkong	Urban	≥15 years	475,900	736			0.88
Wang (49)	2003	2002	Door-to-door	WHO	Heilongjiang Ningxia, Henan, Shanxi, Jiangsu	Both	All	10,151 11,622 12,452 10,273 11,118 55,616	82 99 59 60 87 387			7.00
Ding (50)	2004	2002.06–2002.08	Door-to-door	ILAE	Shanghai	Rural	All	10,777	65	5,392	28	6.03
Chen (14)	2006	2001.01–2001.12	One-stage detection method	ILAE	Taiwan	N/A	≥30 years	13,663	52	5,014		3.81
Chen (51)	2007	2001.12–2007.06	Not mentioned	Local	Ningxia	Rural	All	3,786,000	2,907		1,767	0.77
Liu (52)	2007	2005.05–2005.06	Door-to-door	WHO	Sichuan	Rural	All	2,642,000	1,229			0.47
Ren (53)	2007	2005.02–2005.03	Door-to-door	ILAE	Yunnan	Rural	All	4,465	16	2,434		3.58
Huang (9)	2008	2002.11–2003.10	Door-to-door	WHO	Ningxia	Rural	All	8,316	69	4,000	38	8.30
Zhao (54)	2008	2006.09–2006.10	Door-to-door	ILAE	Tibet	Rural	All	14,822	37	6,926	19	2.50
Wang (55)	2009	2000.11, 2004.11	Door-to-door	ILAE	Shanxi	Rural	All	10,285	61			5.93
Zhao (56)	2009	2009.07	Door-to-door	Local	Tibet	Both	All	14,811	60			4.05
Meng (57)	2010	2008	Door-to-door	ILAE	Jilin	Rural	All	310,402	1,233		650	3.97
Zhao (58)	2010	2008.05–2008.09	Door-to-door	ILAE	Tibet	Rural	All	7,669	180		114	23.47
Yang (59)	2011	2007–2010.06	Door-to-door	ILAE	Hebei	Rural	All	2,066,000	4,902			2.37
Zhao (60)	2011	2010.05–2010.06	Door-to-door	ILAE	Shanghai	Urban	All	106,573	513	55,417	301	4.81
Li (61)	2012	2008.10–2010.12	Door-to-door	WHO	Hebei	Rural	All	20,138	138		76	6.65
Liu (62)	2012	2003	Door-to-door	WHO	Tibet	Both	≥3 years	6,968	50	3,509	22	7.18
Pi (63)	2012	2010.06–2010.10	Door-to-door	ILAE	Hunan	Both	All	32,059	143	16,959	90	4.46
Song (64)	2012	2005.07–2011.06	Door-to-door	WHO	Shandong	Both	All	850,900	640	424,900	342	0.75
Wu (13)	2012	2006.04	Clue investigation	Local	Anhui	Rural	All	898,000	787			0.88
Chang (65)	2013	2012	Door-to-door	ILAE	Jiangsu	Urban	All	19,860	34	9,976	19	1.71
Guo (66)	2013	2012	Door-to-door	WHO	Shandong	Both	All	251,492	335	136,749	185	1.33
Hu (11)	2014	2007–2009	Clue investigation	WHO	Sichuan	Rural	>2 years	3,541,319	6,547	1,835,668	3,606	1.85
Li (67)	2014	2010–2011	Not mentioned	Local	Gansu	Rural	≥2y	2,533,529	4,026		2,304	1.59
Zhang (68)	2020	2014.12–2016.12	Door-to-door	Local	Hubei	Rural	All	1,738,936	8,834			5.08

*Numbers listed in parenthesis immediately after the author's surnames are related to the corresponding references in the reference list.

### Epilepsy prevalence in provincial regions

**Figure 2:**
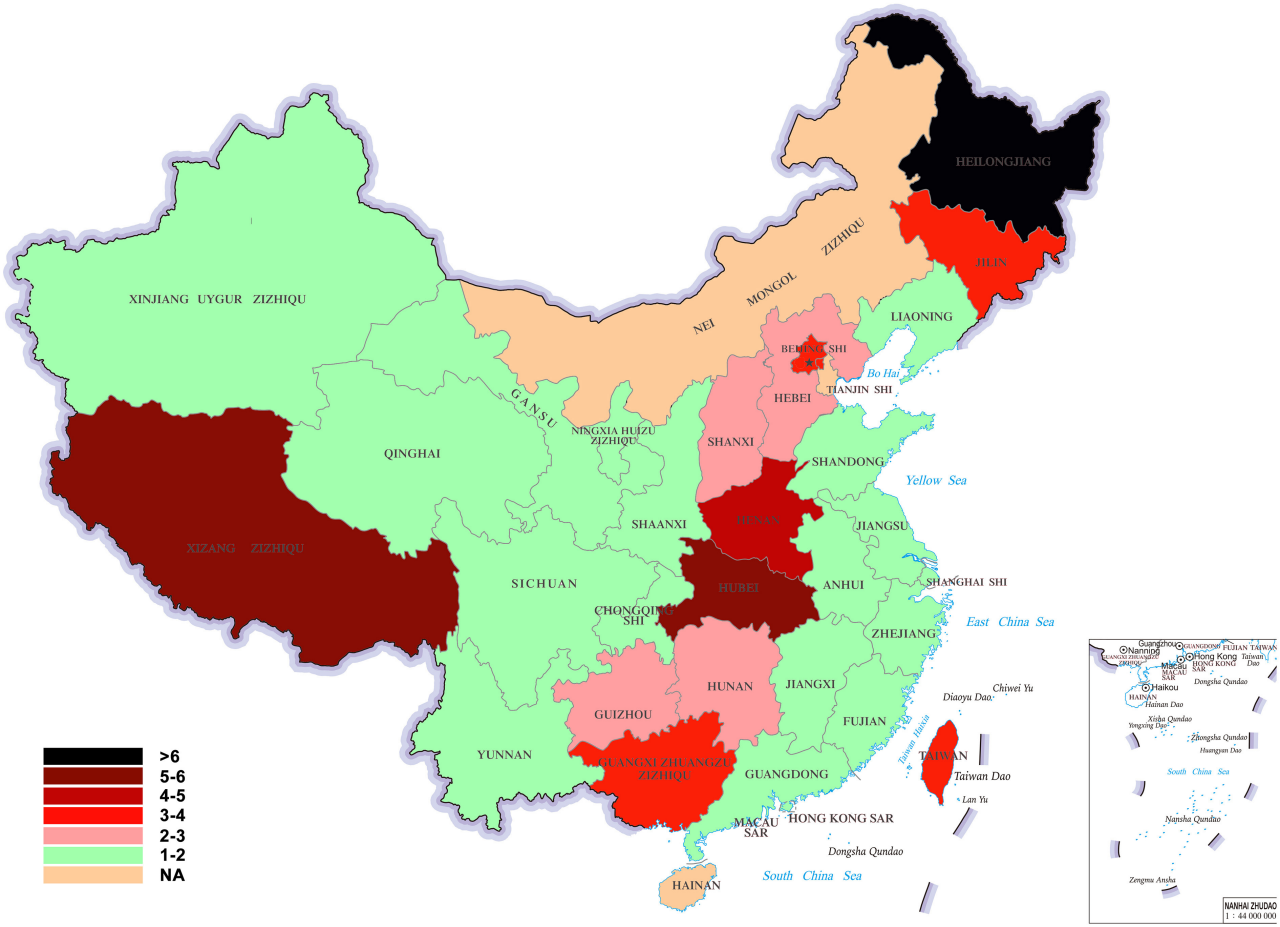
A color-coded map of China showing the prevalence of epilepsy [(case/sampling size) × 1,000‰] in the 33 provincial regions with different color representing different prevalence. In this map, the epilepsy belt was highlighted with deeper colors.

Table 2 showed the epilepsy prevalence in the 29 provincial regions of China in rank order. The average prevalence of epilepsy was 1.68 per 1,000 in the whole China. The prevalence of epilepsy was highest in Heilongjiang (807.8 per 100,000 population) followed by Tibet (Xizang Zizhiqu; 532.4 per 100,000 population), and lowest in Fujian (20.5 per 100,000 population) and Jiangxi (20.2 per 100,000 population). The difference in epilepsy prevalence between the highest and the lowest provincial regions was more than 39 times. Figure 2 was a color-coded map of China showing the prevalence of epilepsy in the 29 provincial regions with different color representing different prevalence.

**Table 2:** Epilepsy prevalence in 29 provincial regions of China.

**Province**	**Sample**	**No. of cases**	**Prevalence (per 1,000)**	**Urbanization (%)**	**GDP per capita (/10,000 US Dollar)**	**Less than 15 years old (%)**	**Medical Insurance per capita (/Yuan, RMB)**
Heilongjiang	10,151	82	8.07	60.10	0.65	11.95	620
Tibet	75,693	403	5.32	31.14	0.66	N/A	1,361
Hubei	1,738,936	8,834	5.08	59.30	1.01	15.92	438
Henan	12,452	59	4.74	51.71	0.76	30.84	374
Jilin	310,402	1,233	3.97	57.53	0.84	11.99	448
Beijing	11,493	44	3.83	86.50	2.11	10.60	3,605
Taiwan	13,663	52	3.81	78	2.50	N/A	N/A
Guangxi	11,201	38	3.39	50.22	0.84	21.71	298
Hebei	2,133,136	5,074	2.38	56.43	0.72	16.83	379
Shanxi	74,278	161	2.17	58.41	0.69	17.09	337
Guizhou	10,173	20	1.97	47.52	0.63	28.10	319
Hunan	933,029	1,710	1.83	56.02	0.80	17.62	325
Liaoning	66,595	109	1.64	68.10	0.88	11.42	876
Jiangsu	170,991	278	1.63	69.61	1.74	13.00	933
Gansu	2,569,934	4,086	1.59	47.69	0.47	18.16	344
Hongkong	475,990	736	1.55	100	4.87	N/A	N/A
Xinjiang	20,236	31	1.53	47.2	0.75	21.86	774
Shanghai	1,327,448	1,994	1.50	88.10	2.04	8.6	3,691
Shandong	2,578,190	3,840	1.49	61.18	1.15	15.74	594
Qinghai	131,003	194	1.48	54.47	0.72	19.71	697
Sichuan	6,354,187	8,240	1.30	52.29	0.74	16.97	480
Guangdong	1,212,692	1,490	1.23	70.70	1.32	16.89	687
Yunnan	84,375	100	1.19	47.81	0.56	20.73	364
Shaanxi	207,304	246	1.19	58.13	0.96	14.70	428
Zhejiang	202,595	211	1.04	68.90	1.50	13.21	1,038
Ningxia	3,840,304	3,230	0.84	58.88	0.82	22.34	692
Anhui	1,004,335	820	0.82	54.69	0.72	17.98	297
Fujian	107,154	22	0.21	65.80	1.38	15.46	559
Jiangxi	108,715	22	0.20	56.00	0.69	21.88	270
Total	25,796,655	43,359	1.68				

### The epilepsy belt

The 12 provincial regions that met our criteria for a region of high epilepsy prevalence included Heilongjiang, Shanxi, Tibet, Hubei, Henan, Jilin, Beijing, Taiwan, Hebei, Guangxi, Guizhou, and Hunan, and constituted a near continuous belt. The prevalence of epilepsy in the epilepsy belt was per 331.9 per 100,000 population compared with 125.3 in regions outside the belt (*P* < 0.05). Except for Tibet and Taiwan, this belt located along the Daxing'an, Taihang, Wu and Xuefeng Mountains, which is the division between the second step and the third step of China. This line-shaped belt was interrupted by the Inner Mongolia, in which there has been no epilepsy prevalence survey.

According to the 2018 China Census, the national population (excluding Taiwan, Hong Kong, and Macao) was 1.395 billion, of whom 592.47 million (44.00%) resided in the epilepsy belt, and 753.78 million (56.00%) outside the belt. The epilepsy belt covered 34.76% (3.337/9.6 million km^2^) of China's territory. The overall prevalence of epilepsy in China was 1.68 per 1,000, but the prevalence of epilepsy in the epilepsy belt was 3.32 per 1,000 compared with 1.25 per 1,000 in other regions (*P* < 0.001). The prevalence of epilepsy in the belt was more than 2 times of that in other regions.

### Prevalence of epilepsy in different provincial regions over time

**Figure 3:**
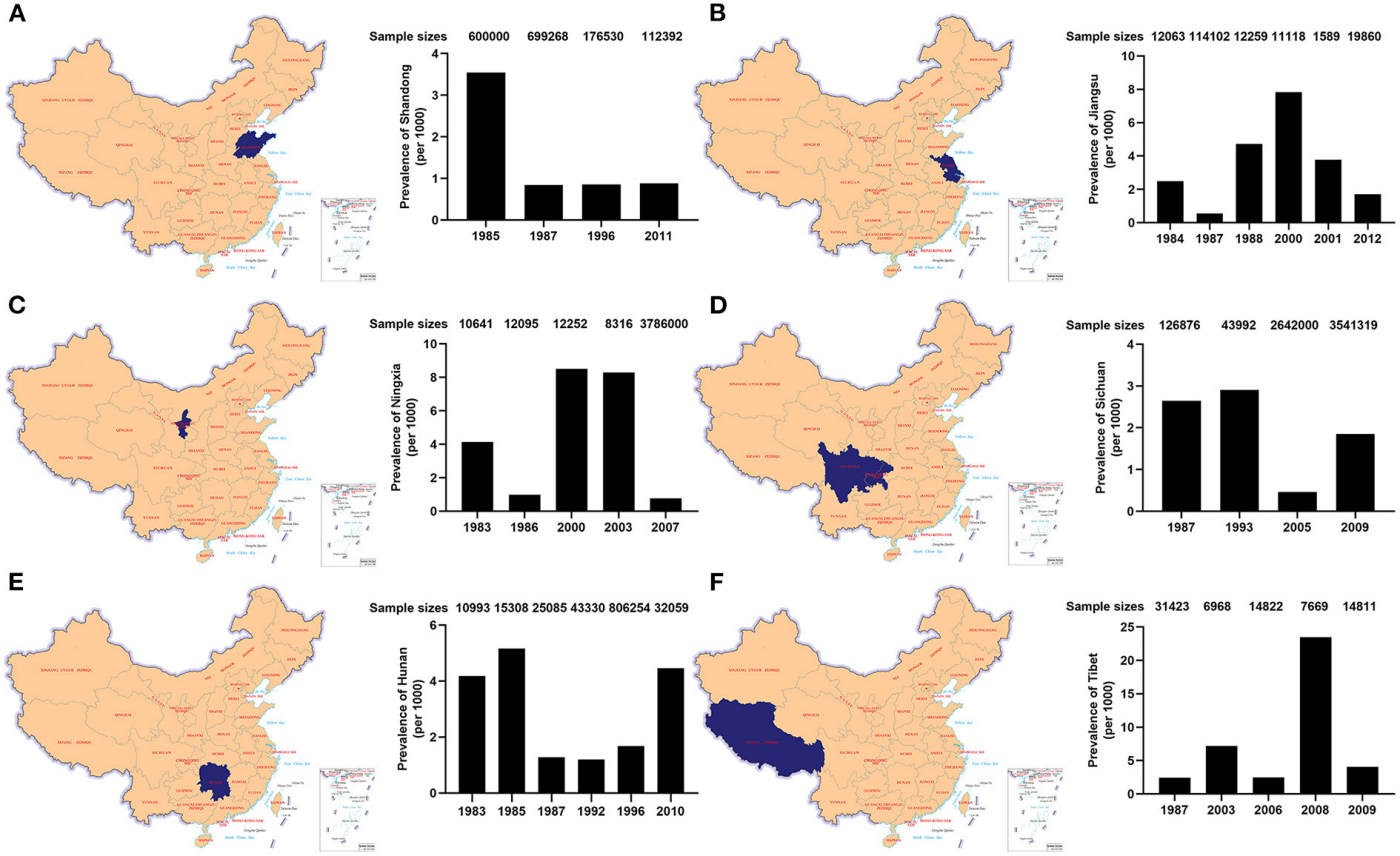
Prevalence of epilepsy in 6 provincial regions over time, **(A)**, Shandong, **(B)**, Jiangsu, **(C)**, Ningxia, **(D)**, Sichuan, **(E)**, Hunan, and **(F)**, Tibet (Xizang Zizhiqu), respectively.

Figure 3 showed epilepsy prevalence of six provincial regions in different years. In Shandong Province, the prevalence of epilepsy was 3.54 per 1,000 in 1985, and declined to 0.84 per 1,000 in 1987, stayed at 0.86 per 1,000 in 1996 and 0.88 per 1,000 in 2011. In Jiangsu Province, the epilepsy prevalence was lowest in 1987 (0.55 per 1,000) and highest in 2000 (7.83 per 1,000), and declined to 3.78 per 1,000 in 2001 and 1.71 per 1,000 in 2012. In Ningxia Province, the prevalence of epilepsy was 4.13 per 1,000 in 1983, and declined to 0.99 per 1,000 in 1986, increased to 8.52 per 1,000 in 2000, maintained at 8.30 per 1,000 in 2003, downed to 0.77 per 1,000 in 2007. In Sichuan Province, the epilepsy prevalence was 2.65 per 1,000 in 1987 and 2.91 per 1,000 in 1993, dropped to 0.47 per 1,000 in 2005, and climbed up to 1.85 per 1,000 in 2009. In Hunan Province, the epilepsy prevalence was 4.18 per 1,000 in 1983, increased to 5.16 per 1,000 in 1985, and fell to around 1.28 per 1,000 during 1987 and 1996, but rose at 4.46 per 1,000 in 2010. In Tibet, the prevalence of epilepsy changed dramatically in the period of 1987 to 2009. It was 2.42 per 1,000 in 1987, 7.18 per 1,000 in 2003, 2.50 per 1,000 in 2006, 23.47 per 1,000 in 2008, and 4.05 per 1,000 in 2009. However, the dramatic difference of epilepsy prevalence over time might be partially caused by variable sample sizes, distinct sampling regions, diverse diagnostic criteria and even sampling bias in these studies conducted in different years.

### Possible risk factors of epilepsy within and outside the epilepsy belt

**Figure 4:**
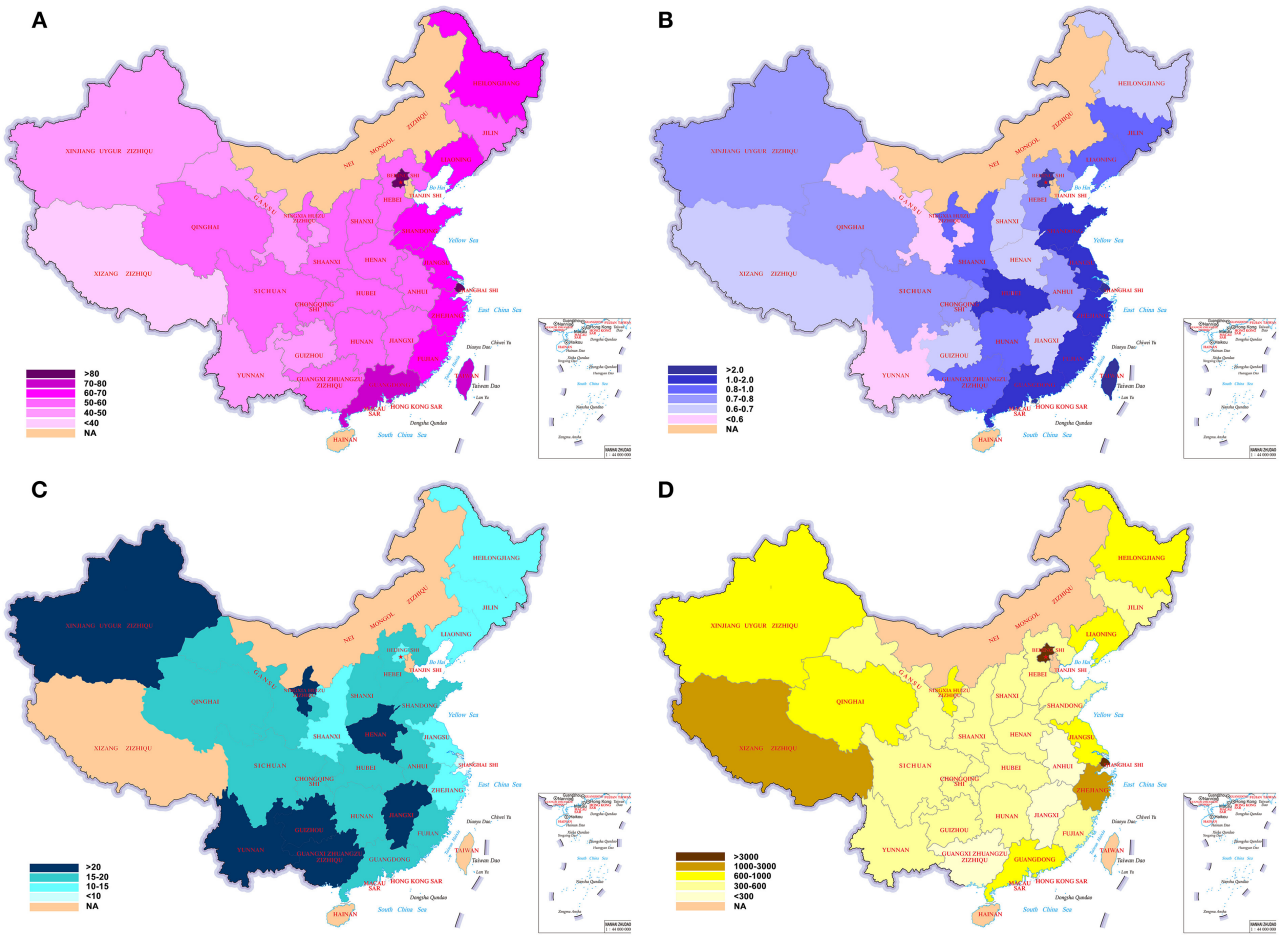
The color-coded maps of possible risk factors for epilepsy in the 33 provincial regions with different color representing different rate. **(A)** Urbanization rates (%), **(B)** GDP per capita (/10,000 US Dollar), **(C)** percentage of <15 years old (%), **(D)** per capita medical insurance (/Yuan, RMB) in 33 provincial regions. There was no significant difference of the urbanization rate, GDP per capita, medical insurance per capita, and percentage of <15 years old between regions in and outside the epilepsy belt (*P* > 0.05), and none of them was correlated with the regional epilepsy prevalence (*P* > 0.05).

Table 2 and Figure 4 also showed the data of urbanization rate, GDP per capita, percentage of <15 years old and per capita medical insurance in 33 provincial regions. There was no significant difference of the urbanization rate, GDP per capita, medical insurance per capita, and percentage of <15 years old between regions in and outside the epilepsy belt (*P* > 0.05), and none of them was correlated with the regional epilepsy prevalence (*P* > 0.05).

## Discussion

It is estimated that there are over 70 million epilepsy patients in the world (2). The death risk of epilepsy patients is 2–3 times of the death risk of general population. In addition to the high mortality, epilepsy also induces disability, while great majority of patients with epilepsy are young patients, which bring severe burden to the families and society. Approximately 80% of epilepsy patients live in low-income and middle-income countries. In China, although the death caused by epilepsy has decreased by 34.8% from 1990 to 2010 (from 187,000 to 122,000), and the DALYs has also decreased from 1,723,100 to 1,597,400, the prevalence of life time epilepsy was found significantly higher at present than in the past (2.42 per 1,000 in 1990 *v.s*. 8.43 per 1,000 in 2015, in the 30–34 age group) (69).

Prevalence rate plays an important role in the secondary and tertiary prevention of diseases. The epilepsy prevalence is higher in China than in developed countries, and was found growing in the past decades, which indicates that it is necessary to improve the health service system in China (70). In this study, we extracted data from 60 studies providing data on the prevalence of epilepsy between 1978 and 2020 in 29 of 33 provincial regions of China. Our study showed that the overall prevalence of epilepsy in China was 1.68 per 1,000. Among the 29 provincial regions, 12 of them, including Heilongjiang, Shanxi, Tibet, Hubei, Henan, Jilin, Hebei, Beijing, Guangxi, Taiwan, Guizhou, and Hunan possessed higher epilepsy (3.32 per 1,000 in average) prevalence than the overall epilepsy prevalence.

In order to investigate the possible reasons of unbalanced epilepsy prevalence in difference regions of China and even the causes of the epilepsy belt, we surveyed the factors reported to be related with epilepsy prevalence, including poverty, young age and countryside (2). Surprisingly, there was no significant difference in urbanization rate, GDP per capita, medical insurance per capita or percentage of <15 years old between the regions with higher epilepsy prevalence and with lower epilepsy prevalence. And none of these factors was correlated with the regional epilepsy prevalence. As we all know, to the worldwide extent, the incidence of epilepsy is higher in children and adolescents than in adults. However, the epilepsy prevalence increases along with age grows, peaks at 25–35 age group and 45–50 age group, and declines after 55 (2). In this study, more specific age groups might lead to positive results, however, majority of those enrolled publications had no specific age groups. Moreover, in recent decades, since the growing population aging leads to increased incidence of cerebral vascular diseases and neurodegenerative disorders, the incidence of epilepsy is growing in elderly people (69), which might be one of the reasons that no differences of young age percentage was found between epilepsy belt and regions outside the belt. In some researches, epilepsy was found tending to affect poor population and rural area (71). However, the data of a large-scale epidemiological investigations conducted in 1980s in urban area of six cities and rural area of 21 provincial regions, revealed that the prevalence of epilepsy in rural area was 3.6 per 1,000, while the epilepsy prevalence in urban area was 4.4 per 1,000 (41), and in our study, data from the whole country sustained this result, the urbanization rate was not correlated with the regional epilepsy prevalence. The GDP per capita and medical insurance per capita, which could reflect the economics and medical conditions in one region, were assumed to be correlated with the regional prevalence of epilepsy, based on the previous researches (2, 71). However, the distinct results from our study provided new evidence to the study of risk factors of epilepsy.

Interestingly, 10 out of the 12 provincial regions constituting the epilepsy belt located along the division between the second step and the third step of China. The highest prevalence was found in Heilongjiang Province (8.08 per 1,000), which occupied the east side of the Daxing'an mountains. China's mainland consists of plains, basins, plateaus, foothills, and mountains, in which rugged plateaus, foothills and mountains form a topographic feature higher in the West and lower in the East like a three-step ladder. The highest step (first step) is formed by the Qinghai-Tibet Plateau at the average height of over 4,000 m, with the Kunlunshan range, Qilianshan range and Hengduan mountain chain as the division between the first and the second step. On the second step, there are large basins and plateaus, most of them are at the height of 1,000–2,000 meters. The second and the third step are divided by the Daxing'an range, Taihang range, Wu and Xuefeng Mountains. The third step is dotted with the lower mountains and foothills, with altitudes of 500–1,000 m (7). Beside of the height, the temperature, humidity (humidity, rainfall), sunshine time, percentage of ethnic minorities, housing area per capita, number of hospital beds per capita, number of doctors per capita, health expenditure, habits and customs, are diverse in these three steps (7). It might be possible that the unique features of the geographic conditions and of the population lived at the division of the second step and the third step contributed to the higher prevalence of epilepsy. The difference of height, temperature, sunshine time, and socio-cultural characteristics mentioned above should be enrolled in during future researches.

Several limitations should be emphasized when interpreting the results of this study. First, retrieving epilepsy prevalence rates from multiple epidemiological surveys may generate bias because of discrepancies in sampling and other methodology. The geographical distribution of the sampling sites of the 60 enrolled studies was uneven. Although the population density in China is uneven, much denser in south China than north China (eg, >700/km^2^ in Jiangsu vs. <10/km^2^ in Xinjiang) (6), the samples size/population was lower in the less populated regions of northern China than in southern China and, in particular, there was no sampling in the Inner Mongolia. Besides, some of the researches focused on urban regions and some of them surveyed in cities covering urban and rural areas, others only investigated in the rural area. In addition, the enrolled studies were conducted during 1978 and 2020, not a specific time point. As the data showed, epilepsy prevalence may change over time in a given region. For instance, in Hunan, the prevalence of epilepsy changed dramatically in the period of 1983 to 2010. It was 4.18 per 1,000 in 1983, 5.16 per 1,000 in 1985, 1.28 per 1,000 in 1987, 1.20 per 1,000 in 1992, 1.68 per 1,000 in 1996, and 4.46 per 1,000 in 2010 (23, 27, 28, 39, 42, 63). However, the dramatic difference of epilepsy prevalence over time might be partially caused by variable sample sizes, distinct sampling regions, diverse diagnostic criteria and even sampling bias in these studies conducted in different years. What's more, some researches focused on a specific age group such as <14 years old (30, 33), and some researches only surveyed a specific epilepsy type (11, 52), both could lead to bias. And majority of the enrolled researches did not include specific age groups, only mentioning all age range or children, based on these data, it is impossible to investigate the relationship between specific age groups and epilepsy prevalence.

## Conclusions

In conclusion, we have identified an epilepsy belt of high prevalence across 12 provincial regions located along the geographical division between the second step and the third step of China. The underlying reasons forming this epilepsy belt should be investigated further for population-based epilepsy prevention.

## Data availability statement

The original contributions presented in the study are included in the article, further inquiries can be directed to the corresponding authors.

## Ethics statement

Ethical review and approval was not required for the study on human participants in accordance with the local legislation and institutional requirements. Written informed consent from the participants' legal guardian/next of kin was not required to participate in this study in accordance with the national legislation and the institutional requirements.

## Author contributions

LW and ZY: data curation, writing—original draft preparation, and funding acquisition. YL: data curation, software, and visualization. ML: project administration and visualization. SL: formal analysis. HM: validation. ZQ: formal analysis, methodology, and funding acquisition. YJ: conceptualization, supervision, writing—review and editing, and funding acquisition. All authors read and approved the final manuscript.

## Funding

This study was supported by the National Natural Science Foundation of China [grant numbers: 81471181, 81870933, and 81701471], the Guangdong Provincial Medical Science and Technology Research Fund [A2021406], the Guangzhou Medical and Health Science and Technology Project [20191A011083 and 20211A011080], the Science and Technology Planning Project of Panyu District [2019-Z04-11], and the Opening Lab Program of Guangzhou Medical University [0506308]. The funding sources had no involvement in the design of the study and collection, analysis, interpretation of data, and in writing the manuscript.

## Conflict of interest

The authors declare that the research was conducted in the absence of any commercial or financial relationships that could be construed as a potential conflict of interest.

## Publisher's note

All claims expressed in this article are solely those of the authors and do not necessarily represent those of their affiliated organizations, or those of the publisher, the editors and the reviewers. Any product that may be evaluated in this article, or claim that may be made by its manufacturer, is not guaranteed or endorsed by the publisher.

## Abbreviations

YLDs: years lived with disability
DALYs: disability-adjusted life-years
CNKI: China National Knowledge Infrastructure
WHO: World Health Organization
ILAE: International League against Epilepsy.
